# Microbiome Structure of the Aphid *Myzus persicae* (Sulzer) Is Shaped by Different Solanaceae Plant Diets

**DOI:** 10.3389/fmicb.2021.667257

**Published:** 2021-07-05

**Authors:** Baoyu He, Xiaoyulong Chen, Hong Yang, Tomislav Cernava

**Affiliations:** ^1^Guizhou Provincial Key Laboratory for Agricultural Pest Management of the Mountainous Region, Guizhou University, Guiyang, China; ^2^College of Tobacco Science, Guizhou University, Guiyang, China; ^3^Institute of Environmental Biotechnology, Graz University of Technology, Graz, Austria

**Keywords:** insect microbiome, Solanaceae, *Nicotiana tabacum*, *Solanum melongena*, *Capsicum annuum*

## Abstract

*Myzus persicae* (Sulzer) is an important insect pest in agriculture that has a very broad host range. Previous research has shown that the microbiota of insects has implications for their growth, development, and environmental adaptation. So far, there is little detailed knowledge about the factors that influence and shape the microbiota of aphids. In the present study, we aimed to investigate diet-induced changes in the microbiome of *M. persicae* using high-throughput sequencing of bacterial 16S ribosomal RNA gene fragments in combination with molecular and microbiological experiments. The transfer of aphids to different plants from the Solanaceae family resulted in a substantial decrease in the abundance of the primary symbiont *Buchnera*. In parallel, a substantial increase in the abundance of *Pseudomonas* was observed; it accounted for up to 69.4% of the bacterial community in *M. persicae* guts and the attached bacteriocytes. In addition, we observed negative effects on aphid population dynamics when they were transferred to pepper plants (*Capsicum annuum* L.). The microbiome of this treatment group showed a significantly lower increase in the abundance of *Pseudomonas* when compared with the other Solanaceae plant diets, which might be related to the adaptability of the host to this diet. Molecular quantifications of bacterial genera that were substantially affected by the different diets were implemented as an additional verification of the microbiome-based observations. Complementary experiments with bacteria isolated from aphids that were fed with different plants indicated that nicotine-tolerant strains occur in Solanaceae-fed specimens, but they were not restricted to them. Overall, our mechanistic approach conducted under controlled conditions provided strong indications that the aphid microbiome shows responses to different plant diets. This knowledge could be used in the future to develop environmentally friendly methods for the control of insect pests in agriculture.

## Introduction

Microorganisms are ubiquitous in the environment and often associated with eukaryotic hosts. During the last decades, it has been shown that microorganisms in plants and animals often fulfill important functions (Van der Ent et al., 2009; Fuchs, 2010). Individual plants and animals often harbor more than 1,000 different microbial species; some of them are crucial determinants for their host’s health and productivity because they can modulate metabolism, improve pathogen defense, and increase resource uptake (Van Der Heijden et al., 2008; Etalo et al., 2018; Alavi et al., 2020; Matsumoto et al., 2021). Insects, analogous to other animals, also have a close relationship with their microbiota; it was previously shown to have a substantial impact on insect ecology and various implications for evolutionary host development (Lewis and Lizé, 2015). Targeted studies have provided evidence that the composition of the insect microbiome is shaped by both geographic location and diet (Guo et al., 2019; Xu et al., 2019; Ma et al., 2021). Symbionts are found ubiquitously in insects among a variety of other microbes with as yet unknown functions. They can play various beneficial roles, such as promotion of growth and defense against natural enemies (Lee et al., 2017; Oliver and Perlman, 2020). Moreover, they support the adaptability of insects to adverse environmental conditions (Dunbar et al., 2007).

Aphids are sap-sucking insects that belong to the globally widespread insect order Hemiptera. Over 4,000 different aphid species were described within this order so far (Dixon et al., 1987). Therein, over 250 species are considered to be among the most destructive pests of cultivated plants (Blackman and Eastop, 1984). Aphids can negatively affect their host plants in different ways. They can damage plants *via* direct and indirect mechanisms; the latter is especially due to transmitted viruses. It is currently assumed that aphids can vector more than 200 viral diseases, which accounts for over 30% of all relevant plant viruses (Brault et al., 2010). In addition, aphids often served in the past as models to study microorganism–insect interactions (Oliver et al., 2010). The bacterial genus *Buchnera*, which includes various symbiont species that can be obligate for their hosts, was found to supply vitamins and essential amino acids that are not taken up through their plant diets, such as methionine and tryptophan (Birkle et al., 2002; Akman Gündüz and Douglas, 2009). In return, the aphid can also provide distinct compounds to its symbionts, which they cannot synthesize due to evolutionary losses of distinct biosynthetic genes (Brinza et al., 2009). Their interaction is therefore obligate and mutualistic in a way that neither partner can reproduce in the absence of the other. *Buchnera* cells are commonly distributed in the cytosol of specialized host cells around the aphid gut; they are often subjected to changes during their host’s lifecycle (Simonet et al., 2016). In some insects, *Buchnera* was shown to be enriched before adulthood and to gradually decrease in adult insects (Simonet et al., 2018). In addition to the obligatory *Buchnera* symbionts, a broad range of secondary symbionts was previously discovered in aphids. Some of them are known to manipulate host reproduction, whereas others are involved in a mutualistic interplay that can increase host survival or fecundity, such as *Serratia symbiotica*, *Hamiltonella defensa*, *Regiella inseticola*, *Rickettsia*, *Rickettsiella*, *Spiroplasma*, *Wolbachia*, *Arsenophonus*, and *Fukatsuia symbiotica* (Scarborough et al., 2005; Russell and Moran, 2006; De Clerck et al., 2015; Ayoubi et al., 2020). Among aphid species, *Myzus persicae* (Sulzer) is one of the most destructive, cosmopolitan, and generalist agricultural pests. It can cause substantial damage to more than 400 plant species (Quaglia et al., 1993). Some subspecies are specifically adapted to Solanaceae plants, which are economically the third most important plant family, consisting of approximately 2,700 plant species (Olmstead and Bohs, 2006; Tapia et al., 2008). Especially the cultivation of pepper (*Capsicum annuum* L.), eggplant (*Solanum melongena* L.), and tobacco (*Nicotiana tabacum* L.) is often affected by *M. persicae*, which can substantially reduce the amount and quality of harvested plant products. Nowadays, the control of *M. persicae* is mainly based on chemical control; however, insecticides have brought serious resistance problems as a result of their extensive use. We aimed at further expanding the knowledge related to factors that shape the aphid microbiome by conducting feeding experiments under controlled conditions. We assume that this knowledge could provide an extended basis for the development of a new control strategy for *M. persicae*. Previous studies with a similar aim found potential implications of the sampling location and host plant; however, the shaping capacity of the latter on the aphid’s microbiota remained to be explored in more detail (Xu et al., 2019). This especially applies to host changes with highly different secondary metabolite profiles. In the present study, we hypothesized that the microbiome of aphids undergoes significant changes as they move from an initial host that produces low levels of bioactive compounds to different plants within the Solanaceae family. We selected three plant species that were fed separately to *M. persicae* populations in a controlled environment. After rearing them on cabbage (*Brassica rapa* L. var. *pekinensis*), they were transferred to eggplant, pepper, and tobacco plants. After 2 weeks, we dissected the aphid guts to which bacteriocytes are attached and extracted the total community DNA. Following a targeted amplification of bacterial 16S ribosomal RNA (rRNA) gene fragments and high-throughput sequencing, the data were subjected to an explanatory bioinformatics approach. From a long-term point of view, our findings may facilitate the development of microbiome management approaches targeting symbiotic bacteria of aphids to minimize their destructive impact on crops.

## Materials and Methods

### *Myzus persicae* Rearing and Sample Preparation

Plants used to feed aphids were cultivated from seeds to provide controlled conditions, especially in terms of agrochemical residues that might affect the microbiota. Cabbage seeds (*B. rapa* L. var. *pekinensis*) were obtained from Guizhou Debang Agricultural Products Co., Ltd. (Guiyang, Guizhou; location: N26°57′83.42″, E106°71′34.78″), eggplant seeds (*S. melongena L.* cv. Zilong No. 8) and pepper seeds (*C. annuum* cv. Gui Yan No. 13) from Guizhou Lifeng Zhongye Co., Ltd. (Guiyang, Guizhou, China), and tobacco seeds of the cultivar MS K326 from Yuxi Zhong Yan Seed Co., Ltd. (Yuxi, Yunnan; location: N24°19′54.32″, E102°31′44.95″). For their cultivation, soil, perlite, and vermiculite were mixed in a 3:3:1 ratio and irrigated with water after the respective seeds were added. They were kept in an incubator at 25°C, 66% relative humidity, and 16-h/8-h day/night cycles for around 10 days until the seeds started to germinate. At the seedling stage, they were transferred to larger pots (11.5 × 9.5 cm) with the same soil mixture as mentioned earlier. The pots were then placed into mesh cages (35 × 35 × 28 cm). One plant was placed into each mesh cage with 12 replicates for each Solanaceae plant diet. For the control group, aphids were kept for the same duration on a cabbage diet in 12 additional mesh cages. When the plants reached stable growth, adult *M. persicae* aphids reared on cabbage were transferred together with cabbage leaves cut from the plants into the mesh cages with the Solanaceae plants to allow a natural transition; the cabbage leaves were subsequently removed from the containers. A complementary experiment with the same setup and six replicates per plant species was conducted to assess the population dynamics of *M. persicae* on the plant diets that were implemented. Observations and counts were made on the first day of transfer from cabbage to Solanaceae plants and continued for 14 days. The average number of aphids was obtained every day and used to construct a graph showing the population dynamics. The temperature and humidity were monitored with an EL-USB-2 device (Lascar Electronics). During the whole feeding experiment, the temperature ranged between 18.5 and 33°C, while the relative humidity ranged between 47 and 98% (Supplementary Figure 1). After 2 weeks on the respective diets, the adult aphids were removed for dissections. For surface sterilization, *M. persicae* was submerged in 75% ethanol for 10 s; the step was repeated in a different tube and followed by transfer into sterile water to remove the residual ethanol. Then, the aphids were placed into a phosphate-buffered saline buffer to prepare them for dissection. Disposable sterile syringe needles (0.3 × 13 mm; Zhejiang Kangdelai Medical Equipment Co., Ltd.) and tweezers (ST-11; Shenzhen Deer Fairy Technology Co., Ltd.) were used to remove the guts, to which also bacteriocytes are attached, under a stereoscope (Olympus SZ2-ILST). In the first step, the heads of the adult aphids were clamped with tweezers. Then, using sterile disposable syringe needles, the abdomens of the aphids were cut open. For each sample, 50 guts from aphids kept in the same mesh cage were collected in a sterile 1.5-ml tube. This was replicated 12 times for each plant diet that was administered in a separate mesh cage. All samples were stored on ice during the dissection process. In total, 48 composite samples, each consisting of 50 aphid guts from separate mesh cages, were obtained and stored at −80°C before total community DNA extraction. The whole experiment procedure is shown schematically in Supplementary Figure 2.

### Total Community DNA Extractions From Aphid Guts

All gut samples were processed with a DNA extraction kit (Fast DNA SPIN Kit for soil; MP Biomedicals, Solon, OH, United States) to extract the total community DNA. The frozen samples were directly transferred into the extraction vials provided in the kit mentioned earlier to avoid contaminations during handling. Subsequently, all total community DNA extractions were conducted according to the manufacturer’s protocol. The extracts were photometrically analyzed with a Nanodrop 2000 device (Thermo Fisher Scientific, Wilmington, DE, United States) to quantify the DNA and verify its quality; 12 biological replicates were obtained with sufficient DNA concentrations for the subsequent steps. The total community DNA extracts were stored at −20°C until further processing.

### Barcoding and High-Throughput Sequencing of 16S Ribosomal RNA Gene Fragment Amplicons

The DNA samples mentioned earlier were sent to a sequencing company (Novogene Co., Ltd., Beijing, China) for next-generation sequencing that targeted the hypervariable region 4 (V4) of bacterial 16S rRNA genes. The samples were amplified with the primers 515f (5′GTGYCAGCMGCCGCGGTAA) and 806r (5′GGACTACHVGGGTWTCTAAT) according to the Earth Microbiome Project protocol^1^ with sample-specific barcodes and Illumina sequencing adaptors. The following polymerase chain reaction (PCR) program (95°C for 5 min to denature the DNA, 30 cycles at 96°C for 60 s, 78°C for 5 s, 54°C for 60 s, 74°C for 60 s, and 10 min at 74°C for a final extension) was used for the generation of amplicons according to a method described before (Thompson et al., 2017). During the PCR amplification, specific peptide nucleic acid (PNA) oligomers were added to the PCR mix to prevent the amplification of mitochondrial (mPNA) or plastidial (pPNA) RNA from eukaryotic origin (Lundberg et al., 2013). The PCR blockers were obtained from PNA Bio Inc. (Newbury Park, CA, United States). Negative controls were included in each PCR reaction. They did not result in visible products and were thus not subjected to sequencing to avoid misassignments due to “index hopping” in low-quantity DNA samples (Van der Valk et al., 2020). High-throughput sequencing was conducted by Novogene (Beijing, China) on the Illumina PE250 platform that produces 2 × 250-bp paired-end reads.

### Bioinformatic Processing of the 16S Ribosomal RNA Gene Fragment Library

The reads were assigned to samples by demultiplexing them according to their unique barcode sequences. All demultiplexed paired-end reads were imported into QIIME2 2019.10 (Bolyen et al., 2019) and quality-filtered using the q2-demux plugin followed by denoising with DADA2 (Callahan et al., 2016) (with the q2-dada2 plugin) to summarize sequence variants (SVs) and to generate a filtered feature table as well as representative sequences. The chimeras were filtered from the table, and taxonomy was assigned to amplicon sequence variants (ASVs) using the q2-feature classifier (Bokulich et al., 2018) in combination with “Greengenes 13_8 99% ASVs” reference sequences (McDonald et al., 2012). Subsequently, the determination of alpha and beta diversity was performed using the QIIME 2 core diversity metrics and group significance tests using the q2-diversity plugin (Faith, 1992) after samples were rarefied to 66,246 reads per sample. The feature table was split into four separate tables according to the sample group (cabbage-fed, pepper-fed, eggplant-fed, and tobacco-fed). For visualization, the feature table was exported from QIIME2, and barplots were generated with a cut-off of 0.1% abundance. Significant differences in the occurrence of distinct ASVs between the control and each treatment were calculated using the R (version 4.03) package edgeR (version 3.30.3) (Robinson et al., 2010).

### Verification of *Pseudomonas* and *Buchnera* Enrichment in the Samples

To confirm changes in the abundance of the genera *Pseudomonas* and *Buchnera* when *M. persicae* was fed with different plant diets, a quantitative PCR (qPCR)-based approach was used. The same samples used to generate the amplicon library were also used to quantify *Pseudomonas* and *Buchnera* in the DNA extracts. All samples were adjusted to a concentration of 2 ng/μl according to quantifications with a Nanodrop 2000 (Thermo Fisher Scientific, Wilmington, DE, United States) to account for the differences in the extraction efficiency and subsequently used for molecular quantifications with a CFX96 Real-Time System (Bio-Rad Laboratories, Hercules, CA, United States). Each sample was analyzed with four technical replicates. The primer pair used for *Pseudomonas* quantification was Pse434F (5′-ACTTTAAGTTGGGAGGAAGGG-3′) and Pse665R (5′-ACACAGGAAATTCCACCACCC-3′) (Pereira et al., 2018). The primer pair used for *Buchnera* quantification was dnaK2F (5′-GATTGTCTTCGGCTGTTG-3′) and dnaK2R (5′-GTCACTCCTTTATCACTTGG-3′). In addition, the aphid’s elongation factor 1α (using primers AWRT002F 5′-CTGATTGTGCTGTGCTTATTG-3′ and AWRT002R 5′-CAAGGTGAAAGCCAATAGAGC-3′) (Jiang et al., 2013) was included in the quantifications as a reference gene. The total reaction volume was 20 μl and contained 10-μl PowerUp SYBR Green Master Mix (Applied Biosystems, Vilnius, Lithuania), 1-μl DNA template, 1-μl (5-nM) forward primer, 1-μl (5-nM) reverse primer, and 7-μl sterile ddH2O. The PCR cycling conditions for *Pseudomonas* quantification included an initial denaturation at 95°C for 5 min, followed by 40 cycles of 95°C for 20 s, 60°C for 20 s, and 72°C for 25 s. The PCR cycling conditions for *Buchnera* quantification included an initial denaturation at 95°C for 5 min, followed by 45 cycles at 95°C for 20 s, 57°C for 20 s, and 72°C for 25 s. For quantification of the aphid’s elongation factor 1α, the initial denaturation was conducted at 95°C for 5 min, followed by 40 cycles at 95°C for 20 s, 59°C for 20 s, and 72°C for 25 s. A melting curve (68–95°C) was obtained in the final step for all three approaches. The cycle threshold was used as a reference to validate differences in the abundance of *Pseudomonas* and *Buchnera* in *M. persicae* grown on different plant diets.

### Isolation of Bacteria From Guts of *M. persicae* and Susceptibility Testing Toward Nicotine

Aphids were randomly collected from the four host plants and dissected as described earlier to obtain aphid 50 guts per sample. After grinding the guts using an automated tissue grinding device (BBI Life Sciences, Shanghai, China) for 5 s, the guts were transferred into 10% nutrient broth and incubated at 30°C on a shaker at 180 rpm/min for 12 h. Subsequently, a micropipette was used to transfer 10-μl bacterial suspension after dilution with 0.85% NaCl (10^–1^–10^–3^ dilutions) to Petri dishes with LB agar, nutrient agar, and R2A agar. The plates were cultivated at 30°C until the appearance of bacterial colonies. For *Pseudomonas* isolations, *Pseudomonas* isolation broth was used to incubate 50 guts that were dissected from cabbage- and Solanaceae-fed aphids. Incubations were performed at 30°C on a shaker at 180 rpm/min for 12 h. Diluted suspensions (10^–1^–10^–3^ dilutions) were then transferred to *Pseudomonas* CFC selective agar (Qingdao Hope Bio-Technology Co., Ltd.) with *Pseudomonas* CFC selective agar supplement (Qingdao Hope Bio-Technology Co., Ltd.) and to King’s B medium (Qingdao Hope Bio-Technology Co., Ltd.). They were cultivated at 30°C until bacterial colonies appeared, which were subsequently purified. The bacterial genomic DNA extraction kit (Beijing Solarbio Science & Technology Co., Ltd.) was used to extract the DNA of all isolates before PCR amplification of 16S rRNA gene fragments for taxonomic identification with the primer pair 27F (5′-AGAGTTTGATCCTGGCTCAG-3′) and 1492r (5′-GGTTACCTTGTTACGACTT-3′). The PCR amplification comprised an initial cycle at 95°C for 2 min, followed by 34 cycles of denaturation at 95°C for 30 s, annealing at 50°C for 1 min, extension at 72°C for 2 min, and a final extension step at 72°C for 10 min. The length of PCR products was confirmed by 1% gel electrophoresis, and the PCR products were sent to Sangon Biotech (Shanghai, China) for Sanger sequencing. Based on the obtained 16S rRNA gene fragment sequences, phylogenetic placement was conducted. The candidate taxa for the phylogenetic analysis included all isolates from aphid guts and such with high similarities in BLAST searches within the National Center for Biotechnology Information (NCBI) nucleotide database. To construct a phylogenetic tree with those sequences, multiple alignments were performed using Clustal W (Thompson et al., 1994). Subsequently, molecular phylogenetic analyses based on neighbor-joining algorithms were created with MEGA7 (Kumar et al., 2016). Bootstrap values were calculated with 1,000 replications for the neighbor-joining method.

All isolates were additionally subjected to susceptibility testing toward nicotine, a bioactive metabolite characteristic of Solanaceae plants. Bacteria were cultivated on nutrient medium and R2A medium (for slow-growing bacteria) to obtain single colonies. Sterile toothpicks were used to transfer single colonies to the nutrient broth and liquid R2A medium, followed by incubation for 16 h at 30°C and 180 rpm/min. Subsequently, the bacterial suspensions were diluted to an optical density measured at a wavelength of 600 nm of 0.1 in a liquid R2A liquid medium. Aliquots of 5 ml were transferred into glass test tubes (18 × 180 mm) with 0, 2, 4, and 8 g/l nicotine passed through a 0.22-μm PES filter (Tianjin Keyilong Lab Equipment Co., Ltd) for sterilization. The glass test tubes were incubated in a shaker at 30°C and 160 rpm/min. The optical density measured at a wavelength of 600 nm was then recorded each 6 h for 48 h with the Multiskan GO instrument (Thermo Fisher Scientific, Vantaa, Finland) to monitor bacterial growth in a nicotine-supplemented medium.

### Statistical Analyses

Statistical tests for the microbiome analyses were performed using the QIIME 2 and R studio (R version 4.03) software packages. The significance of the differences in alpha diversity was tested with the implemented Kruskal–Wallis test and for the beta diversity with the analyses of similarities test in the QIIME 2 pipeline. The R package edgeR was implemented to identify differently abundant genera between the treatments (log_2_ fold change > 2 and log_2_ fold change < −2; *P* < 0.01). The statistical significance of the differences in the qPCR data was assessed with the Kruskal–Wallis test.

## Results

### Alpha and Beta Diversity Analyses of the Aphid Microbiome Under Different Plant Diets

**FIGURE 1:**
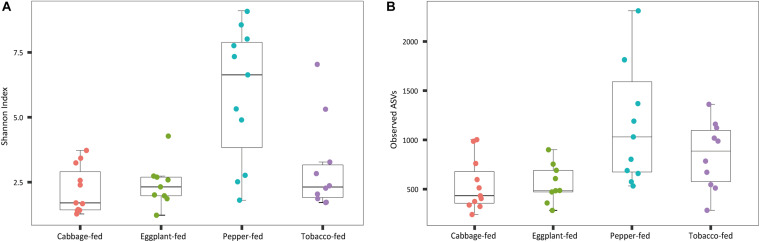
Alpha diversity of bacterial communities in *Myzus persicae* subjected to different plant diets. Diversity was assessed within the QIIME2 pipeline on basis of Shannon index **(A)** and observed operational taxonomic units (ASVs) **(B)** in the normalized dataset. All samples were rarefied to a sequence depth of 66,246 reads before being subjected to diversity analyses.

**FIGURE 2:**
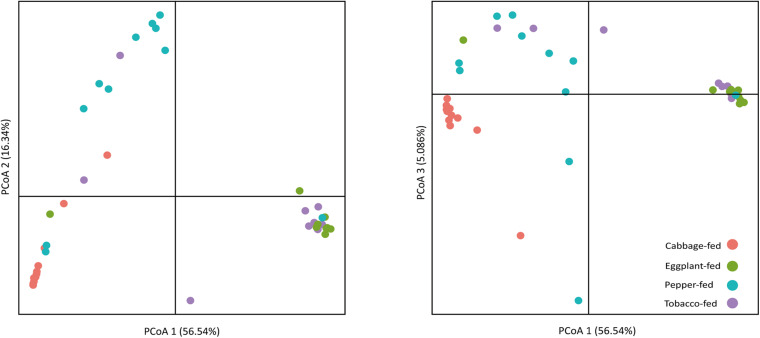
Principal coordinates analysis (PCoA) of bacterial community structure in cabbage-fed and Solanaceae-fed aphids. Three dimensions that explain highest degree of variance were included in visualization. Percentage variance explained is indicated on respective axes. Different colors of dots indicate different sample types. Significance of differences between different bacterial communities was tested with ANOSIM in QIIME 2.

After filtering chimeric, mitochondrial, and plastid sequences, the feature table was reduced from a total read count of 6,200,722 to 5,310,692, representing 20,876 features. Data normalization resulted in a read count of 66,246 per sample; a total number of 20,111 features remained in the dataset. Collapsing features on genus level resulted in 1,607 bacterial genera and 37 archaeal taxa. The Shannon index (Figure 1A) was used to compare the bacterial diversity among the different samples. The microbiomes of cabbage-fed aphids (*H*′ = 2.21 ± 0.27), eggplant-fed aphids (*H*′ = 2.41 ± 0.28), and tobacco-fed aphids (*H*′ = 3.05 ± 0.56) had a significantly lower alpha diversity than pepper-fed aphids (*H*′ = 5.88 ± 0.78; *P* < 0.05). There were no significant differences between the other groups (*P* > 0.05). Similar results were obtained when the number of observed ASVs (Figure 1B) was compared between cabbage-fed aphids (observed ASVs = 544 ± 80), eggplant-fed aphids (obs. ASVs = 560 ± 65), tobacco-fed aphids (obs. ASVs = 845 ± 108), and pepper-fed aphids (obs. ASVs = 1,163 ± 180). Significant differences were found when cabbage-fed aphids and eggplant-fed aphids were compared with pepper-fed aphids (*P* < 0.05), whereas no significance was observed between pepper-fed aphids and tobacco-fed aphids (*P* > 0.05). Beta diversity analyses revealed distinct clustering of sample groups based on Bray–Curtis dissimilarities (Figure 2). Analyses of similarities confirmed highly significant (*R* = 0.5; *P* = 0.001) differences in community composition between the cabbage-fed and the Solanaceae-fed aphids.

### Assessment of the Bacterial Community Structure and Composition

**FIGURE 3:**
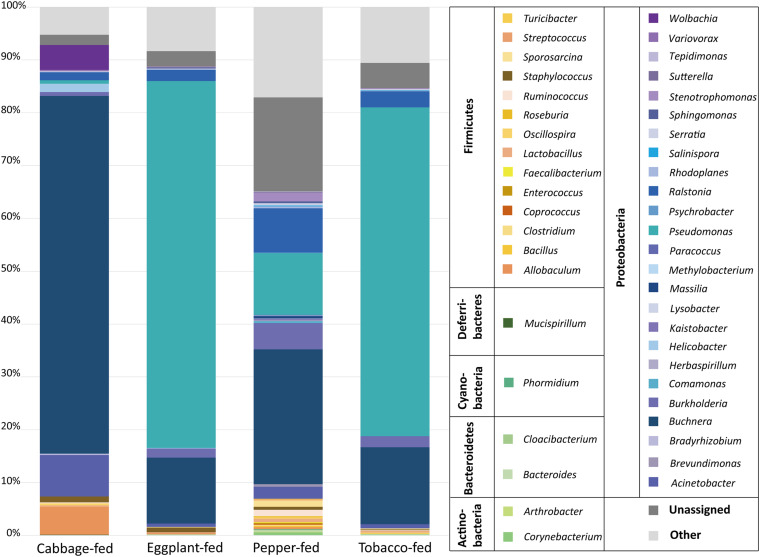
Bacterial community structure of different sample types. Taxonomic classification of highly abundant (>0.1%) members of aphid microbiome on different diets. Taxa with a lower abundance than the implemented threshold were summarized as “other.” Bacterial community structure was visualized up to genus level for each sample type. In addition, all taxa were grouped on bacterial phylum level in legend for better differentiation.

At the bacterial family level, the cabbage-fed aphids were primarily colonized by *Enterobacteriaceae* (68.2%), *Moraxellaceae* (7.9%), *Erysipelotrichaceae* (5.3%), and *Rickettsiaceae* (4.7%; Figure 3). The prevalent bacterial genera were identified as *Buchnera* (67.7%), *Acinetobacter* (7.8%), and *Allobaculum* (5.3%). The eggplant-fed aphids were mainly colonized by the bacterial families *Pseudomonadaceae* (69.5%), *Enterobacteriaceae* (13.4%), *Oxalobacteraceae* (2.4%), and *Burkholderiaceae* (1.8%). *Pseudomonas* (69.4%), *Buchnera* (12.5%), *Ralstonia* (2.1%), and *Burkholderia* (1.7%) were the most common genera in the eggplant-fed aphids. In tobacco-fed aphids, *Pseudomonadaceae* (62.3%), *Enterobacteriaceae* (15.9%), *Oxalobacteraceae* (3.2%), and *Burkholderiaceae* (2.2%) were the prevalent taxonomic groups on the bacterial family level. *Pseudomonas* (62.2%) was the predominant genus, followed by *Buchnera* (14.6%), *Ralstonia* (3.0%), and *Burkholderia* (2.1%). In the pepper-fed aphids, *Enterobacteriaceae* (29.3%) was the most prevalent bacterial family, whereas *Pseudomonadaceae* (11.8%) and *Oxalobacteraceae* (9.3%) were less abundant, followed by *Burkholderiaceae* (5.1%). The predominant genus in pepper-fed aphids was *Buchnera* (25.5%). The second most abundant genus was *Pseudomonas* (11.7%), followed by *Ralstonia* (8.4%), *Burkholderia* (5.0%), *Enterobacteriaceae* (3.2%), *Acinetobacter* (2.3%), and *Stenotrophomonas* (1.7%). Notably, the pepper plant diet not only showed the least increase in the abundances of *Pseudomonas* and the least decrease in the abundance of *Buchnera* but also other changes in the microbiome when compared with the other two Solanaceae plants. Several low-abundant members of the Gram-positive bacterial phylum *Firmicutes* increased under the pepper diet, which reflects the previously observed differences in alpha and beta diversity (Figures 1, 2).

### Identification of Bacteria That Were Significantly Affected by the Diet

**FIGURE 4:**
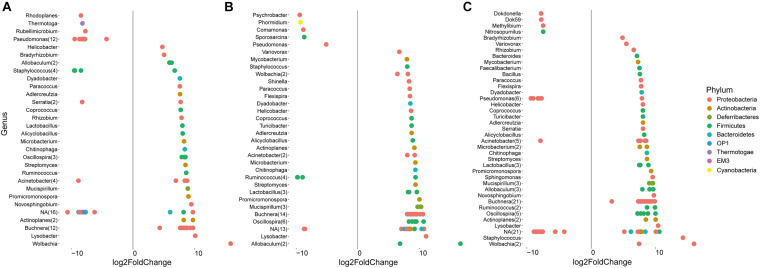
Identification of amplicon sequencing variants (ASVs) that differ significantly in their relative abundance among treatments. Pairwise comparisons were conducted for Solanaceae-fed aphids and cabbage-fed aphids using edegR tool (*P* < 0.01). **(A)** Comparison of cabbage-fed aphids with eggplant-fed aphids; **(B)** comparison of cabbage-fed with pepper-fed aphids; **(C)** comparison of cabbage-fed aphids with tobacco-fed aphids. Taxonomy of ASVs was assigned at bacterial genus level whenever it was possible.

In addition to the general analysis of the microbiome in differently fed aphids, deepening statistical analyses based on edgeR were conducted to identify differentially abundant taxa in each of the treatment groups (Figure 4 and Supplementary Dataset 1). Taxa that were significantly more abundant in eggplant-fed aphids included *Pseudomonas* (12 features), *Staphylococcus* (three features), *Serratia*, *Rhodoplanes*, *Thermotoga*, *Rubellimicrobium*, *Acinetobacter*, and unassigned bacteria (12 features). Forty-six features were shown to be depleted when eggplant-fed aphids were compared with cabbage-fed aphids (Figure 4A). In pepper-fed aphids, *Psychrobacter*, *Phormidium*, *Comamonas*, *Sporosarcina*, *Pseudomonas*, *Ruminococcus* (three features), and unassigned bacteria (three features) were enriched. In contrast, 61 features were enriched in cabbage-fed aphids when compared with pepper-fed aphids (Figure 4B). In the tobacco diet group, 21 features were more abundant than in the cabbage group and assigned to *Dokdonella*, *Dok59*, *Methylibium*, *Nitrosopumilus*, *Pseudomonas* (five features), *Acinetobacter*, and unassigned bacteria (11 features). The remaining 81 features were found to be enriched in cabbage-fed aphids (Figure 4C). When all groups were comparatively assessed, *Pseudomonas* was found to be enriched in all Solanaceae-fed aphids, whereas 21 genera were depleted in those aphids when compared with cabbage-fed aphids. The depleted genera were assigned to *Buchnera*, *Wolbachia*, *Helicobacter*, *Acinetobacter*, *Actinoplanes*, *Adlercreutzia*, *Alicyclobacillus*, *Allobaculum, Chitinophaga*, *Coprococcus*, *Dyadobacter*, *Lysobacter*, *Lactobacillus*, *Microbacterium*, *Mucispirillum*, *Oscillospira*, *Paracoccus, Promicromonospora, Ruminococcus, Staphylococcus*, and *Streptomyces* (Figure 4).

### Quantitative Polymerase Chain Reaction Confirmed Relative Enrichment of *Pseudomonas* and Depletion of *Buchnera*

**FIGURE 5:**
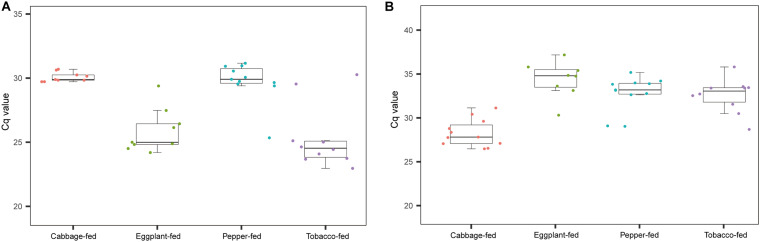
Complementary molecular quantifications of changes in relative abundance of *Pseudomonas* and *Buchnera* in aphids on different plant diets. Enrichment of *Pseudomonas* and depletion of *Buchnera* in Solanaceae-fed aphids was confirmed with a complementary qPCR-based approach and genus-specific detection primers. **(A)** Cq values of *Pseudomonas* in gut of *M. persicae* fed with different plants; **(B)** Cq values of *Buchnera* fed with different plants.

As a complementary approach to the marker gene sequencing analyses, the abundance of *Pseudomonas* and *Buchnera* was confirmed by qPCR targeting these taxa in the total community DNA extracts (Figure 5). The conducted Kruskal–Wallis test for the cycle thresholds indicated that *Pseudomonas* was significantly increased in eggplant-fed aphids (Cq = 25.9 ± 0.6) and tobacco-fed aphids (Cq = 25.4 ± 0.8; *P* < 0.01) when compared with cabbage fed aphids (Cq = 30.1 ± 0.1). However, the difference between pepper-fed aphids (Cq = 29.8 ± 0.5) and cabbage-fed aphids (Cq = 30.1 ± 0.4; *P* = 0.7903) was not significant. *Buchnera* showed a significant decline in eggplant-fed aphids (Cq = 33.9 ± 0.8), pepper-fed aphids (Cq = 32.2 ± 0.6), and tobacco-fed aphids (Cq = 31.9 ± 0.7; *P* < 0.01). The overall results reflected the observations of the microbiome analyses, especially in the substantial reduction of *Buchnera* in Solanaceae-fed aphids. A complementary quantification of the aphid’s elongation factor 1α in total community DNA extracts indicated that the host–microbe DNA proportion was similar among all sample groups (Supplementary Figure 3). Complementary analyses of *M. persicae* population dynamics on different hosts indicated that it was only negatively affected following the transition to pepper plants (Supplementary Figure 4).

### Isolation of Bacteria From *M. persicae* and Their Phylogenetic Placement

**FIGURE 6:**
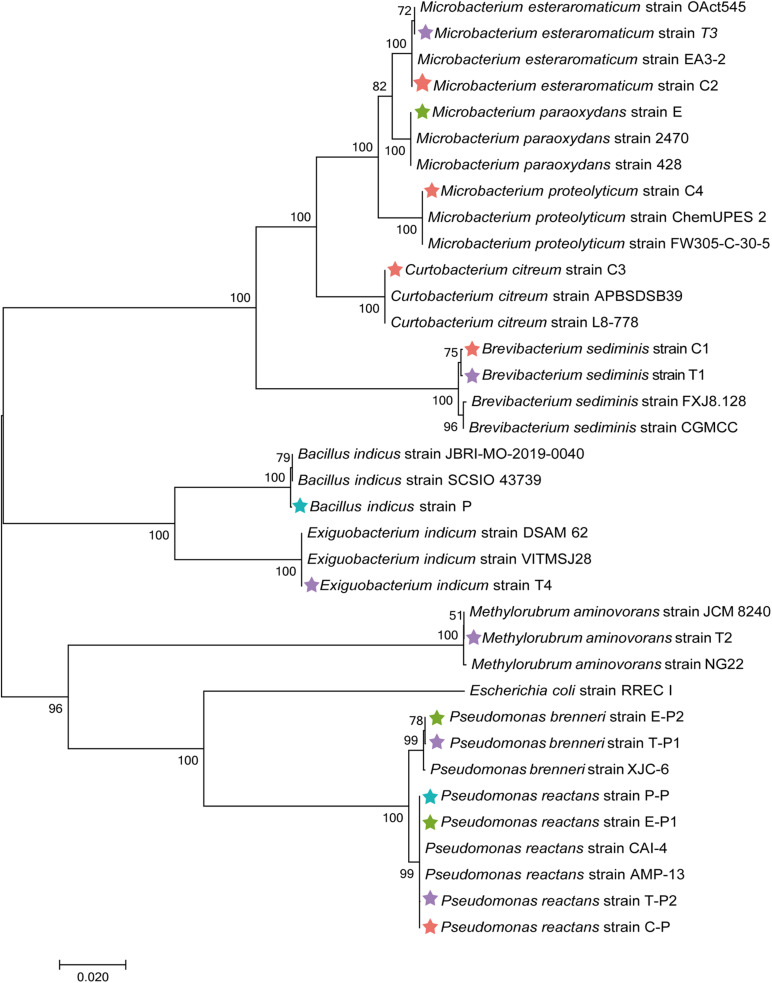
Phylogenetic placement of isolated bacteria based on their 16S rRNA gene fragment sequences. Neighbor-joining method in MEGA7 with 1,000 bootstrapping replications was used to generate phylogenetic tree. Highly matching sequences of bacteria from public nucleotide databases were included; *Escherichia coli* RREC I was used as an outgroup. Isolates from cabbage-fed aphids are labeled with red stars, eggplant-fed aphids with green stars, pepper-fed aphids with blue stars, and tobacco-fed aphids with purple stars.

To obtain complementary insights into the observations of the microbiome analysis, isolation of gut bacteria on different cultivation media was conducted. Guts of *M. persicae* aphids feeding on cabbage, eggplant, pepper, and tobacco were dissected, and a total of 16 isolates was obtained using LB agar, nutrient agar, R2A agar, *Pseudomonas* CFC selective agar, and King’s B medium. Five bacterial strains were isolated from aphids reared on the cabbage diet and assigned to *Curtobacterium citreum*, *Brevibacterium sediminis*, *Microbacterium esteraromaticum, Microbacterium proteolyticum*, and *Pseudomonas reactans.* Six strains were isolated from aphids fed with tobacco and assigned to *B. sediminis*, *Methylorubrum aminovorans*, *M. esteraromaticum*, *Exiguobacterium indicum*, *Pseudomonas brenneri*, and *P. reactans*. Three strains that were respectively assigned to *Microbacterium paraoxydans*, *P. reactans*, and *P. brenneri* were isolated from aphids fed with eggplant. *Bacillus indicus* and *P. reactans* were isolated from aphids that were fed with pepper. The isolates were used for phylogenetic placements based on their 16S rRNA gene fragments (Figure 6). All isolates showed a high similarity to sequences deposited in the NCBI nucleotide database, indicating that the cultivable fraction of the *M. persicae* microbiome consists of bacteria that were previously described.

### Susceptibility Testing of Bacterial Isolates Toward Nicotine

Isolated bacteria from guts of *M. persicae* growing on four plants diets were subjected to specific susceptibility tests. To assess the susceptibility of bacteria toward nicotine, their growth in medium with 0, 2, 4, and 8 g/l nicotine was monitored for 48 h (Supplementary Figure 5). According to the results of a two-factor analysis of variance, the growth rate of all bacterial was significantly reduced with an increasing concentration of nicotine or even completely restrained (*P* < 0.01). *M. aminovorans* strain T2 was not able to grow in any medium supplemented with nicotine. *C. citreum* strain C3, *M. proteolyticum* strain C4, *P. reactans* strain C-P, *E. indicum* strain T4, *P. brenneri* strain T-P1, *P. brenneri* strain T-P2, *M. paraoxydans* strain E2, *P. reactans* strain E-P1, *P. brenneri* strain E-P2, and *P. reactans* strain P-P were not able to grow in medium supplemented with 8 g/l nicotine. Isolates that were able to grow in medium supplemented with 8 g/l nicotine included the *B. sediminis* strain C1 and *M. esteraromaticum* strain C2 that were obtained from cabbage-fed aphids, two isolates from tobacco-fed aphids (*B. sediminis* strain T1 and *M. esteraromaticum* T3), and the *B. indicus* strain P that was isolated from pepper-fed aphids.

## Discussion

By assessing the changes in the bacterial community of *M. persicae* fed with three Solanaceae plants (eggplant, pepper, and tobacco) and cabbage as a reference treatment, we could mechanistically confirm that the microbiome of aphids is influenced by the plant diet. This is in line with recent findings of Xu et al. (2019) that assessed the microbiome of *Aphis gossypii* (Hemiptera: Aphididae) and found clear indications that it was affected by its host plants. In contrast, previous field studies conducted with *M. persicae* feeding on different plants did not show significant differences in the aphid’s microbiome composition (Xu S. et al., 2021). This is most likely due to the described migration of the aphids. In the present study, this factor was excluded by conducting feeding experiments under controlled conditions. The present study provides indications that the microbiome of *M. persicae* can be substantially shaped by the host plant and that these changes take place within a short period. More in-depth studies will be needed in the future to assess whether these changes are temporary or whether they manifest in subsequent generations. For this purpose, targeted studies could be conducted in which aphids are transferred back to the original host plant, followed by microbiome monitoring.

Overall, we found that even if the aphids were fed with different plants, *Buchnera* still remained as the most abundant primary symbiont in *M. persicae* guts and the attached bacteriocytes; however, it was substantially reduced after the transfer to Solanaceae hosts. *Buchnera* is known for its importance as the primary symbiont of various insects and is commonly inherited from mother to offspring (Baumann et al., 1995). We hypothesize that *Buchnera* was substantially reduced after the transition of aphids to the three Solanaceae hosts either because the plants’ secondary metabolites had a negative effect on it or they provided more suitable conditions for its competitors. This observation was confirmed by complementary qPCR analyses, which showed analogous trends when compared with the microbiome results. The implications of nutrient provision by primary symbionts of insects are well known (Moran et al., 2003; Akman Gündüz and Douglas, 2009). It can therefore be assumed that a temporally reduced occurrence of the primary symbiont, at least in the gut and the attached bacteriocytes, does not negatively affect the host. Interestingly, pepper-fed aphids showed a different bacterial community composition than the other two Solanaceae-fed aphids. The most evident difference was a lower increase of the genus *Pseudomonas* represented by one ASV, whereas the other two Solanaceae-fed aphids showed higher increases represented by multiple ASVs. Moreover, *Pseudomonas* was found to occur at very low abundances in cabbage-fed aphids (0.6%), but it substantially increased (maximum observed abundance: 69.4%) after the transition to Solanaceae-based diets. Complementary qPCR-based analyses confirmed a substantial enrichment in tobacco-fed and eggplant-fed aphids, whereas the aphids subjected to the pepper diet showed a comparatively lower enrichment of quantifiable *Pseudomonas* sequences in the total community DNA extracts. This is in line with the microbiome profiles that were generated with the high-throughput sequencing approach. Some *Pseudomonas* species are known to be pathogens (Flury et al., 2016), whereas others have beneficial traits, and some are even commercialized for certain applications in agriculture (Kupferschmied et al., 2013). In addition, various *Pseudomonas* species are associated with crop plants and can occur in different tissues and the plant rhizosphere. Some were found to be important elicitors of induced systemic resistance in plants (Kloepper et al., 2004). When plants are under attack by pathogens or insect pests, they can recruit beneficial pseudomonads for their defense (Lee et al., 2012). A previous study indicated that certain plant-beneficial *Pseudomonas* strains could improve the growth of feeding aphids under certain conditions (Blubaugh et al., 2018). The substantial enrichment of *Pseudomonas* in *M. persicae* observed in the present study may be partially due to uptake from host plants. We could also show that some pseudomonads are naturally occurring in the aphid’s gut, although at substantially lower abundances if they are not subjected to a Solanaceae-based diet. Further future studies will be required to determine the proportion of bacteria transferred from the host plant to the feeding aphids. They will require additional sequencing of the plant leaf microbiome before exposure to aphids. A study by Stavrinides et al. (2009) showed that *Pseudomonas syringae* could use pea aphids as hosts and vectors to infect other plants. *Pseudomonas* was generally shown to be a highly efficient colonizer of insect hosts (Lindeberg et al., 2008). In a recent study, it has been shown that certain members of this genus can change from a commensal to a pathogenic lifestyle in the gut of insects (Xu L. et al., 2021). Our findings indicate that *Pseudomonas* may play a role in the adaption of aphids to new host plants. This observation is reinforced by our complementary analysis of population dynamics, which revealed that only bell pepper plants in which the smallest increases in *Pseudomonas* abundance were found negatively affected aphids. The microbiome of aphids fed with pepper plants was also characterized by an increase of low-abundant members of the bacterial phylum *Firmicutes*. The genera *Sporosarcina* and *Ruminococcus* have significantly enriched these aphids. Members of the genus *Sporosarcina* are occurring in the environment and are known for their bioremediation potential (Achal et al., 2012), whereas *Ruminococcus* is mainly associated with the gut system of mammals (La Reau et al., 2016). Less is known about their potential roles in insects. The underlying mechanisms of the diet-induced microbiome changes and the potentially linked implications for host fitness remain to be explored in more detail in the future because they may provide an exploitable basis for the control of *M. persicae* and other insect pests. In addition to the increased abundance of *Pseudomonas* and decrease of *Buchnera* in Solanaceae-fed aphids, another bacterium that is commonly associated with insects was also subjected to changes. *Wolbachia* is generally known to be less abundant in aphids than the predominant *Buchnera* but can also occur as a symbiont (Augustinos et al., 2011). In the present study, *Wolbachia* followed the same trend as *Buchnera*; a substantial decrease in its relative abundance was observed after the aphid’s transition to Solanaceae plants. Based on the observations of this study, it seems that this reduction also does not negatively affect the host.

As a complementary approach to the bioinformatics analyses, we also aimed to assess parts of the cultivable fraction in the present study to explore potential links to their tolerance of a bioactive metabolite common in the Solanaceae plant family. Nicotine has strong insecticidal properties and is used in different formulations for plant protection (Casanova et al., 2002). It was previously observed that *M. persicae* could adapt to high nicotine concentrations in host plants (Ramsey et al., 2014). Although certain genes are associated with detoxification processes in the aphid, other mechanisms remained unexplored. To assess the potential roles of facultative symbionts isolated from *M. persicae* guts, we conducted tolerance assays with nicotine. Nicotine is generally known to have toxic and pharmacological properties and thus affects a wide range of organisms, including bacteria (Hossain and Salehuddin, 2013; Vieira et al., 2013). We could show that several bacterial strains isolated from aphid guts displayed a high nicotine tolerance under laboratory conditions. Various facultative symbionts are not required for aphid survival, reproduction, and invasion, but they can still play important roles in their host (Oliver et al., 2010). For instance, facultative symbionts can affect the aphid’s adaptability to plants, and they can improve nutrient uptake of their host among various other protective properties (Russell and Moran, 2006; Oliver et al., 2010; Tsuchida et al., 2010; Gauthier et al., 2015). The susceptibility tests toward nicotine showed that the isolated bacteria tolerated the toxic metabolite to different extents. With the increasing nicotine concentrations, the growth rate of all tested bacteria decreased. *M. aminovorans* (basionym: *M. aminovorans*) strain T2 was an exception; it was already inhibited by low nicotine concentrations, although it was isolated from aphids feeding on tobacco. Methylobacteria are nonspore-forming bacteria and form Gram-negative rods that can utilize methanol and often other small hydrocarbons for their growth Urakami et al. (1993). They are commonly found in the atmosphere, soil, and on plant leaves in close spatial proximity to stomata, where some can produce plant-growth-promoting substances (Holland, 1997; Green and Ardley, 2018). We assume that *M. aminovorans* T2 occurred in the aphid gut without having any implications for the host’s adaptability to the host change. Among the other tested isolates, *B. sediminis*, *M. esteraromaticum*, and *B. indicus* strains were shown to tolerate nicotine at up to 8 g/l, but their growth rates were substantially reduced at this concentration. These isolates might be important facultative symbionts that facilitate the adaptation of *M. persicae* to Solanaceae-based diets. Interestingly, *P. brenneri* T-P1 and *P. reactans* T-P2, which were both isolated from tobacco-fed aphids, also tolerated nicotine well to a concentration of 4 g/l. This might be sufficient to facilitate the aphid’s proliferation on the Solanaceae plant diets, but the detailed implications of pseudomonads under these conditions remain to be further explored.

## Conclusion

In summary, our microbiome-guided assessment of bacterial communities in aphids subjected to different diets has shown that a transition to Solanaceae host plants resulted in substantial adaptations of their microbiome. The most evident response was the significant reduction of the primary symbiont *Buchnera* and the simultaneous increase of *Pseudomonas*. Complementary analyses revealed that facultative symbionts might facilitate the aphid’s transition to secondary-metabolite-rich hosts. If the implications are confirmed, then future biotechnological developments could make use of the findings by harnessing pseudomonads that can thrive in insect guts but negatively affect the fitness and propagation of their hosts.

## Data Availability Statement

The 16S rRNA gene fragment amplicon library was deposited in European Nucleotide Archive (ENA; accession number: PRJEB42912) and the 16S rRNA gene sequences of the isolated bacteria were deposited in NCBI Genbank (accession numbers MW564208–MW564219 and MW699619–MW699622).

## Author Contributions

TC and HY conceived the idea and developed the study design. BH performed all laboratory experiments under the supervision of TC, HY, and XC. TC and BH performed the bioinformatic analyses, interpreted the data, and prepared the final visualizations. HY and XC provided valuable inputs related to insect rearing and physiology. TC, HY, and BH wrote the manuscript. All authors reviewed the final version of the manuscript.

## Conflict of Interest

The authors declare that the research was conducted in the absence of any commercial or financial relationships that could be construed as a potential conflict of interest.

## Footnotes

1 www.earthmicrobiome.org
