# Supplementary material for: Microbiome Structure of the Aphid Myzus persicae (Sulzer) Is Shaped by Different Solanaceae Plant Diets
Source: Front Microbiol. 2021 Jul 5;12:667257. doi: 10.3389/fmicb.2021.667257 (PMC8287905; doi:10.3389/fmicb.2021.667257)
Supplement: Supplementary file 2 [file Data_Sheet_1.docx]

**Supplementary Information**

**The Microbiome Structure of the Aphid *Myzus persicae* (Sulzer) is Shaped by Different Solanaceae Plant Diets**

Baoyu He^1^, Xiaoyulong Chen^1,2^, Hong Yang^1,2*^ and Tomislav Cernava^2,3*^

1. Guizhou Provincial Key Laboratory for Agricultural Pest Management of the Mountainous Region, Guiyang, China.
2. College of Tobacco Science, Guizhou University, Guiyang, China
3. Institute of Environmental Biotechnology, Graz University of Technology, Graz, Austria

*corresponding authors: tomislav.cernava@tugraz.at (**TC**) & axyridis@163.com (**HY**)

**Submitted to**: Frontiers in Microbiology

**Short title**: *Myzus persicae* microbiome

**Keywords**: Insect microbiome, Solanaceae, *Nicotiana tabacum*, *Solanum melongena*, *Capsicum annuum*


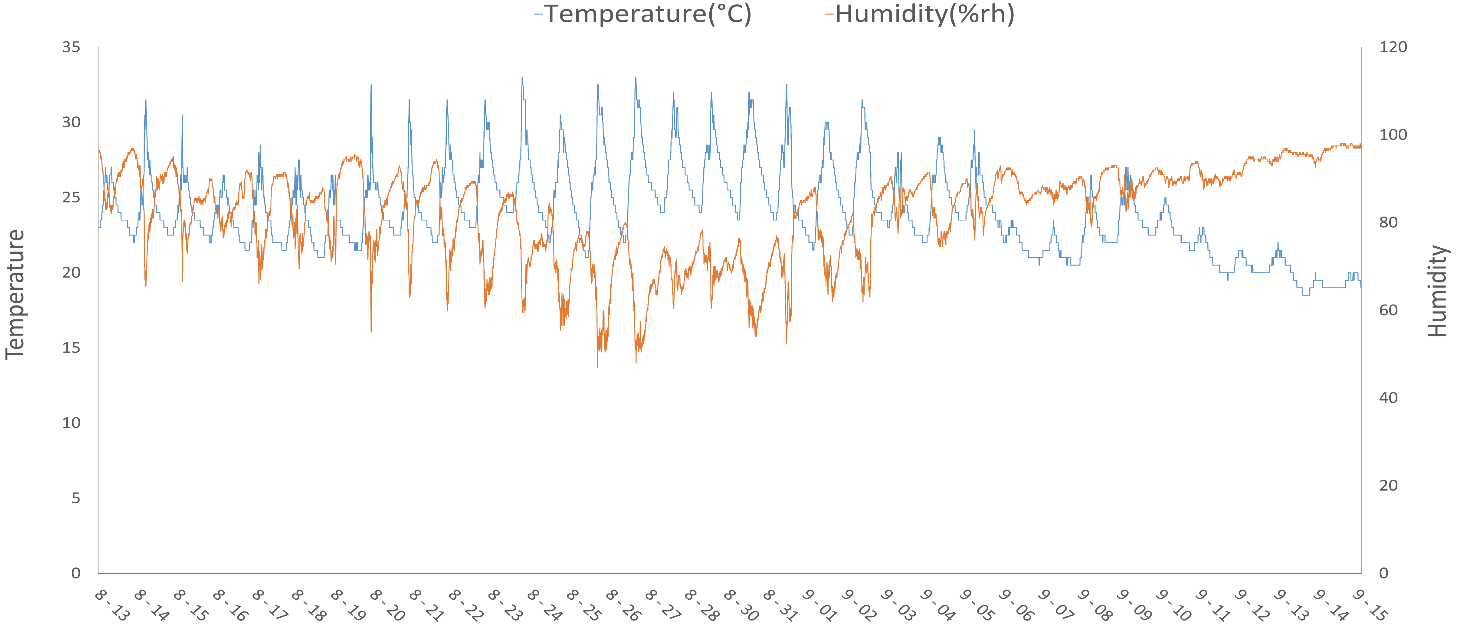


**Figure S1.** Temperature and humidity profiles during the whole duration of the feeding experiments. The parameters were automatically recorded with a EL-USB-2 device (Lascar Electronics).


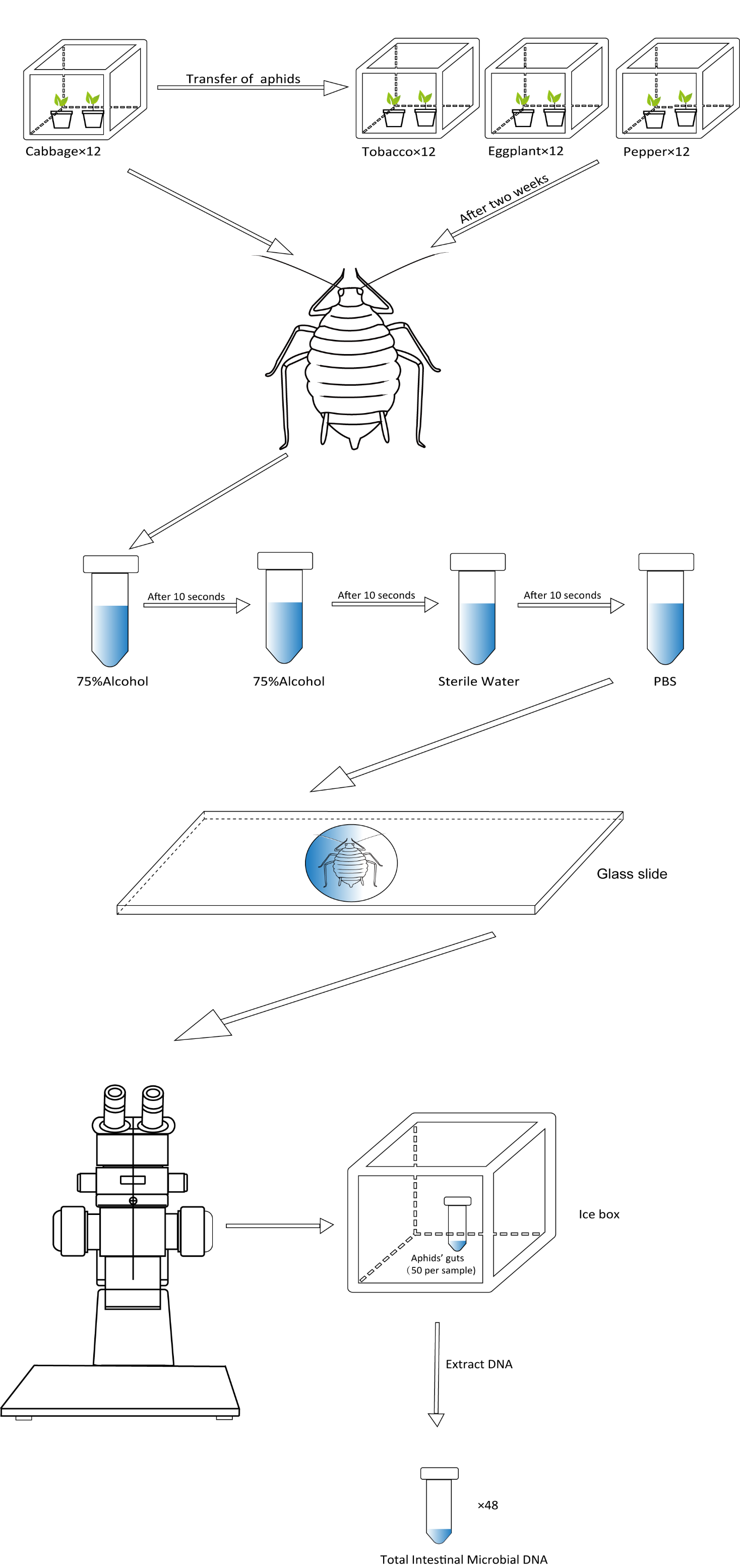


**Figure S2.** Schematic overview of the conducted sample preparations. A total of 50 aphid guts was obtained for each composite sample; this was replicated 12 times for each plant diet.


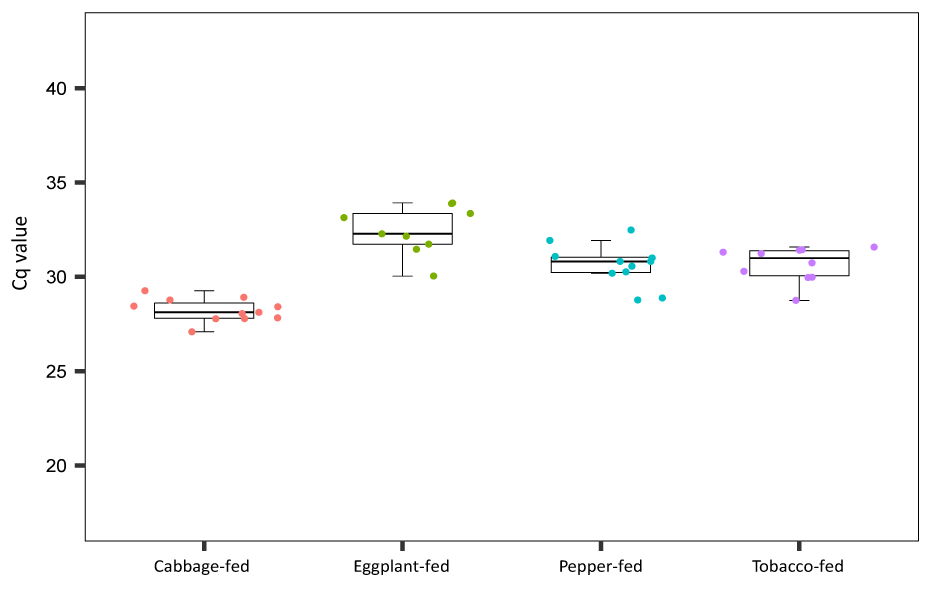


**Figure S3.** Quantification of the elongation factor 1α in *Myzus persicae*. A referece gene of the host was selected to confirm that bacterial DNA was extracted at comparable efficieny for each sample type.

**
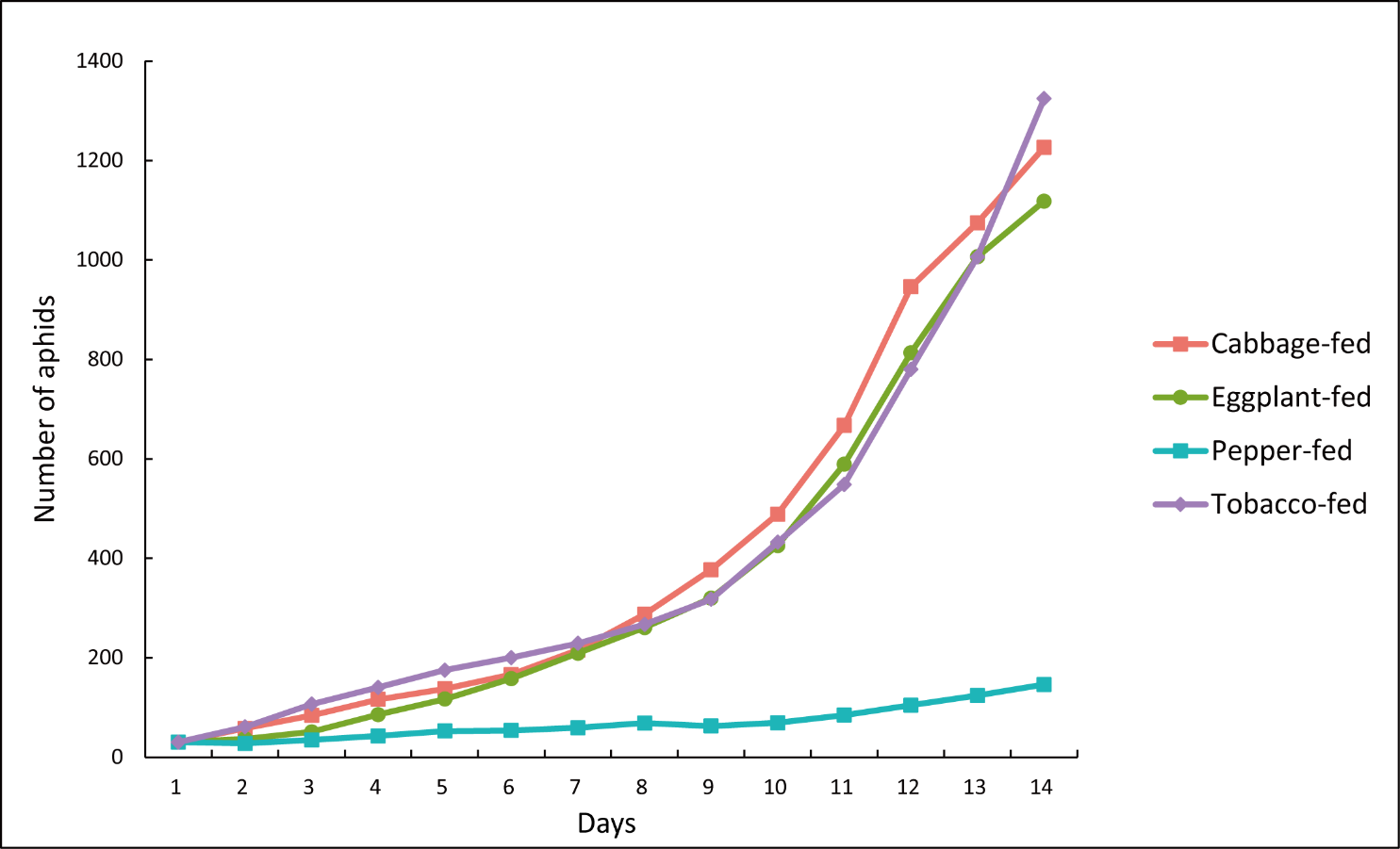
**

**Figure S4.** Effects of host change on aphid population dynamics during an observation period of 14 days. The population size was recorded every two days after the aphids were transferred to the respective host plant.


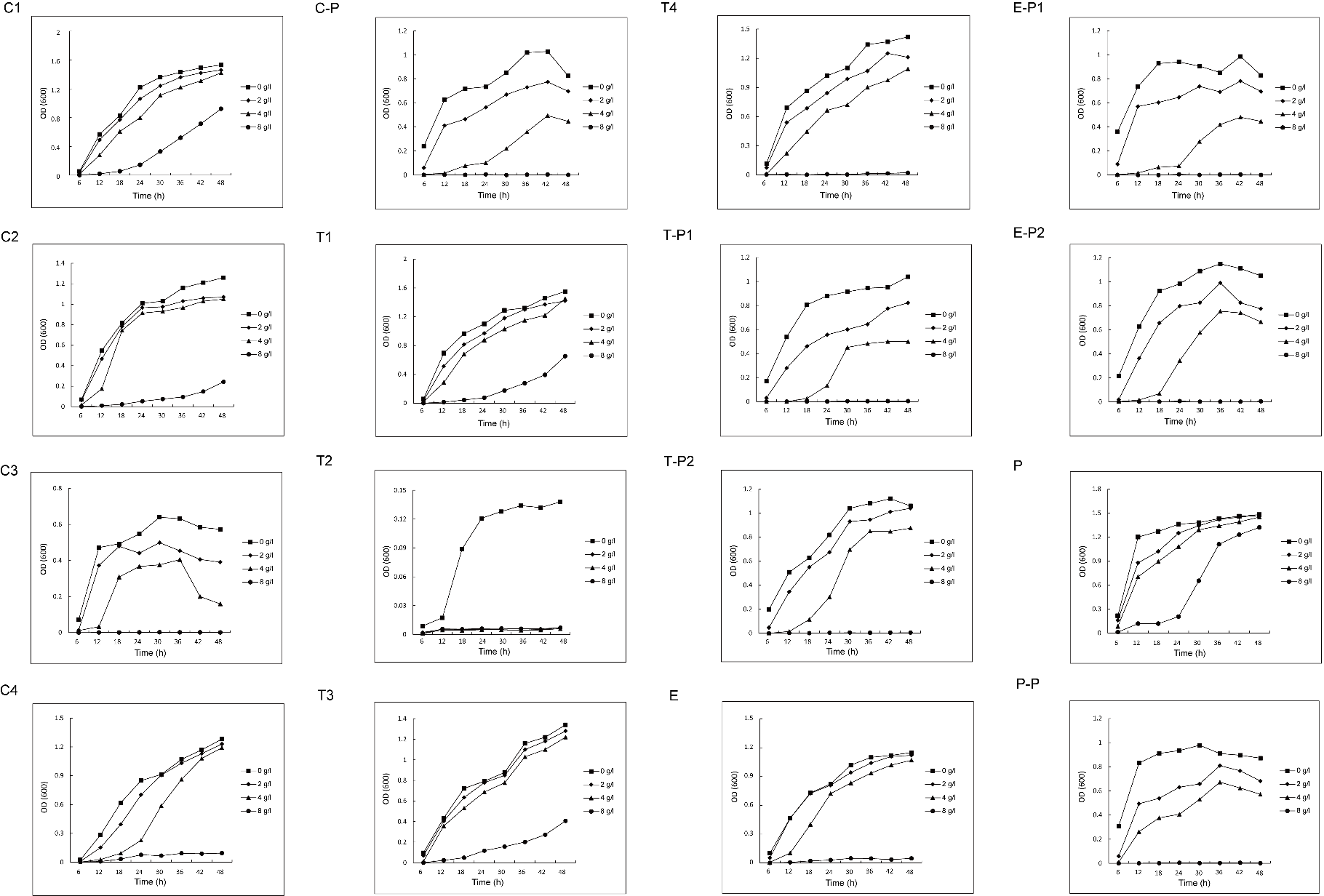


**Figure S5.** Growth experiments with different nicotine concentrations in fluid media. Bacterial strains were isolated from aphids that were reared on different plant diets and tested for their nicotine tolerance at 0 g/l, 2 g/l, 4 g/l and 8 g/l, respectively. Strain names were used as lables for each panel; details related to the taxonomy and phylogenetic placement of the bacterial isolates are included in **Fig. 6**.
